# Dengue virus non-structural protein 1: a pathogenic factor, therapeutic target, and vaccine candidate

**DOI:** 10.1186/s12929-018-0462-0

**Published:** 2018-07-24

**Authors:** Hong-Ru Chen, Yen-Chung Lai, Trai-Ming Yeh

**Affiliations:** 1 The Institute of Basic Medical Sciences, College of Medicine, National Cheng Kung University, Tainan, Taiwan; 2 Department of Medical Laboratory Science and Biotechnology, College of Medicine, National Cheng Kung University, Tainan, Taiwan

**Keywords:** Dengue virus (DENV), Nonstructural protein 1 (NS1), Hemorrhage, Coagulopathy, Vascular leakage, Vaccine

## Abstract

Dengue virus (DENV) infection is the most common mosquito-transmitted viral infection. DENV infection can cause mild dengue fever or severe dengue hemorrhagic fever (DHF)/dengue shock syndrome (DSS). Hemorrhage and vascular leakage are two characteristic symptoms of DHF/DSS. However, due to the limited understanding of dengue pathogenesis, no satisfactory therapies to treat nor vaccine to prevent dengue infection are available, and the mortality of DHF/DSS is still high. DENV nonstructural protein 1 (NS1), which can be secreted in patients’ sera, has been used as an early diagnostic marker for dengue infection for many years. However, the roles of NS1 in dengue-induced vascular leakage were described only recently. In this article, the pathogenic roles of DENV NS1 in hemorrhage and vascular leakage are reviewed, and the possibility of using NS1 as a therapeutic target and vaccine candidate is discussed.

## Background

### General information about dengue

Dengue virus (DENV) is the most common mosquito-borne flavivirus and threatens people in tropic and subtropical areas. The World Health Organization estimates that more than 2.5 billion people representing over 40% of the world’s population are at risk of dengue infection [1]. Dengue virus infections are often asymptomatic or cause a flu-like syndrome with fever and rash. However, a small proportion of cases develop into severe illness, which is termed dengue hemorrhagic fever (DHF). DHF is characterized by vascular leakage, thrombocytopenia, and coagulopathy [2]. Vascular leakage results in hemoconcentration and serous effusions, leading to circulatory collapse, which further develops into life-threatening dengue shock syndrome (DSS) [2]. An estimated 390 million infections occur each year globally, and approximately 960,000 people with severe dengue require hospitalization [1]. Children contribute to a large proportion of the severe disease cases. In 1958, DHF was reported to carry a case fatality rate (CFR) of 13.9% in Bangkok [3]. Even with standardized diagnosis and management, the CFR remained in the range of 0.5–1.7% from 2000–2011 in the Philippines [4]. Despite the high mortality of DHF/DSS, no promising viral-specific drugs or vaccines are available due to the limited understanding of the complicated pathogenic mechanism.

Several hypotheses have been proposed to explain the pathogenesis of DHF/DSS [5]. Among them, antibody-dependent enhancement (ADE) has been proposed to explain why most DHF/DSS cases occur in children who are secondarily infected with a different serotype of DENV from the previous one [6]. Based on ADE, antibodies that are generated by a single DENV infection contribute to lasting homotypic immunity but may permit heterotypic DENV infection. Furthermore, these serotype non-specific antibodies may augment heterotypic virus entry and replication in Fcγ receptor-bearing macrophages, leading to enhanced viremia, antigenemia and cytokine storm [7]. This scenario may also explain why infants who passively acquire maternal anti-dengue antibodies are more likely to develop DHF/DSS following primary infection [8]. However, ADE dose not explain why vascular leakage and hemorrhage occur in DHF/DSS patients. Only when we better understand the molecular mechanisms of DENV pathogenesis can a more effective and specific therapy or vaccine against DHF/DSS be developed. In this review, we focus on the pathogenic roles of DENV non-structural protein 1 (NS1) in the pathogenesis of DHF/DSS. The potential of NS1 as a drug target or vaccine candidate to treat or prevent dengue will be discussed.

### DENV structure

The DENV particle is approximately 500 Å in diameter and includes a positive-sense RNA genome with ~10,700 nucleotides and 3 structural proteins: capsid (C, 100 amino acids), precursor membrane (prM, 75 amino acids), and envelope (E, 495 amino acids) [9]. The capsid protein and the viral RNA genome form a nucleocapsid that buds at the endoplasmic reticulum (ER) in association with 180 copies of prM and E and carries host-derived lipids to form the immature virion [10]. Initially, the immature virion is covered by 60 spikes, each of which is composed of E trimers with associated prM proteins. The maturation process requires the host protease furin, which cleaves prM into the pr and M proteins in the Golgi after the noninfectious virion passes through the cell’s secretory system, which is an acidic environment. This cleavage results in a rearrangement of E to the immature dimer structure, in which E maintains interactions with pr and M [11]. After budding from the cell via exocytosis, the neutral pH of the extracellular environment dissociates E and pr to form mature virions, which are available to infect new cells [11]. In addition to the structural proteins, the RNA genome of dengue virus encodes 7 nonstructural proteins that are essential for viral replication (NS1, NS2A, NS2B, NS3, NS4A, NS4B and NS5).

### NS1 structure, expression and secretion

DENV NS1 is a 48-kDa glycoprotein that is highly conserved among all flaviviruses [12]. NS1 is essential for viral replication with an unknown mechanism that possibly involves interactions with NS4A and NS4B [13, 14]. Initially, NS1 is expressed as a monomer in infected cells. After post-translational modification in the ER lumen, it forms homodimers associated with organelle membranes and the cell membrane [12]. Despite the lack of a transmembrane region, NS1 anchors to the cell membrane through several pathways. The mechanisms are unclear, but anchorage of NS1 to glycosyl-phosphatidylinositol and lipid rafts has been shown [15, 16]. In addition, NS1 is the only protein that is continuously secreted by infected host cells. NS1 is secreted in a hexamer form, which is composed of three dimers with a detergent-sensitive hydrophobic central cavity that carries a cargo of ~70 lipid molecules; the composition is similar to a high-density lipoprotein [17, 18]. This lipid-rich structure may help secreted NS1 attach to the cell membrane by associating with glycosaminoglycans (GAGs) [19]. Due to the similarity between NS1 and high-density lipoprotein, NS1 has been proposed to disrupt the coagulation cascade possibly through interfering with the interaction or biogenesis of endogenous lipoprotein particles [18]. Accumulation of secreted NS1 in DHF/DSS patient sera has been observed during the critical phase [20]. The serum concentration of NS1 in DHF/DSS patients can reach as high as 50 μg/ml, and the concentration is positively correlated with the disease severity [21–23]. During the recovery phase, NS1 is cleared from the circulation by antibody-mediated effects. Because secreted NS1 can interact with complement protein, it was first described as a soluble complement-fixing (SCF) antigen that could promote C4 degradation and in turn possibly protect DENV from complement-dependent lysis [24–26]. Recently, pathogenic roles for secreted NS1 in DHF/DSS have been demonstrated due to its involvement in systemic immunity and endothelial cell activation. In this review, we focus on the molecular mechanisms underlying how NS1 may contribute to vascular leakage, coagulopathy and thrombocytopenia during dengue infection. The possibility of targeting NS1 as a drug and vaccine development target against dengue infection will also be discussed.

## The pathogenic roles of NS1 in vascular leakage

### Pathogenic factors of vascular leakage in dengue pathogenesis

Based upon in vitro data or mouse models, it was once concluded that endothelial cell apoptosis led to vascular permeability during DENV infections and that this was because direct infection of endothelial cells by DENV or damage by antibodies (Abs) against NS1 which can cross-react with endothelial cells [27–34]. However, plasma leakage improves within 1 to 2 days in DHF/DSS patients who receive appropriate fluid resuscitation, and tissue samples from these patients show little structural damage in their vessels. Therefore, apoptosis of endothelial cells induced by DENV infection or anti-NS1 antibodies is not sufficient to support the clinical outcome. As a result, endothelial dysfunction but not apoptosis induced by a dengue-specific factor is currently considered to play a more important role in causing vascular leakage in DHF/DSS [35–37].

### Contribution of the NS1 protein to vascular leakage

DENV NS1-induced vascular leakage has been widely discussed since 2015. A previous study demonstrated that NS1 proteins induced vascular leakage, and applying anti-NS1 antibodies attenuated NS1-induced vascular leakage as well as the mortality rate in mice [38]. However, the mediating receptor of NS1 remains controversial. One study suggested that blocking TLR2 or TLR6 attenuated DENV NS1-induced secretion of TNF-α and IL-6 by peripheral blood mononuclear cells (PBMCs) [39]. TLR6 deficiency also reduced DENV NS1-induced mortality in mice [39]. However, another study demonstrated that NS1-activated TNF-α and IL-1β mRNA expression and IL-6 secretion were attenuated by blocking TLR4 in PBMCs [40]. In contrast, TLR2 inhibition did not alter the effects induced by NS1 in PBMCs [40]. These authors also showed that blocking TLR4 rescued NS1-induced endothelial hyperpermeability, indicating that NS1-induced vascular leakage was mediated by TLR4 [40]. Later, the same group published a short communication to explain that the different results might be caused by contamination of *Escherichia coli*-derived recombinant NS1 with multiple TLR ligands and that TLR4 should be regarded as the real NS1 receptor [41]. However, it has also been shown that DENV NS1 induces similar levels of vascular leakage in TLR4-receptor-deficient mice and wild-type animals, which indicates that NS1-induced vascular leakage can be independent of TLR4 [42]. Taken together, these results suggest that NS1 may contribute to vascular leakage through both TLR4-dependent and independent mechanisms.

In our previous study, we demonstrated that autophagy-mediated junction disruption was involved in DENV NS1-induced vascular leakage, which may explain why vascular leakage in dengue patients is a quick and reversible pathogenic change [43]. NS1-induced macrophage migration inhibitory factor (MIF) secretion is involved in NS1-induced autophagy of endothelial cells [43]. An in vitro study also showed that DENV-infected cells induced MIF secretion, which can cause endothelial hyperpermeability. Furthermore, Mif^−/−^ mice exhibited reduced pathogenesis in a model of severe dengue [44], indicating the importance of MIF in dengue pathogenesis [45]. In fact, several clinical studies have shown that the MIF concentration is elevated in dengue patients [46, 47] and that the MIF concentration is higher in DHF patients who die than in DHF survivors and DF patients [48].

In addition to disrupting endothelial junctions, NS1 also causes vascular leakage by inducing endothelial glycocalyx degradation mediated by heparanase-1 (HPA-1) [42, 49]. The glycocalyx is a thin, negatively charged network consisting of glycoproteins, proteoglycans, and glycosaminoglycans at the luminal side of endothelial cells lining blood vessels throughout the body [50]. To maintain homeostasis, the glycocalyx acts as a barrier that controls numerous physiological processes, such as regulating vascular permeability, preventing the adhesion of leukocytes and blood platelets to the vessel walls [51, 52], mediating shear stress [53, 54], and modulating inflammatory and hemostatic processes. Damage of the endothelial glycocalyx correlates to several vascular pathologies, including ischemia/reperfusion, hypoxia, sepsis, volume overload, diabetes and atherosclerosis [50, 55].

**Fig. 1:**
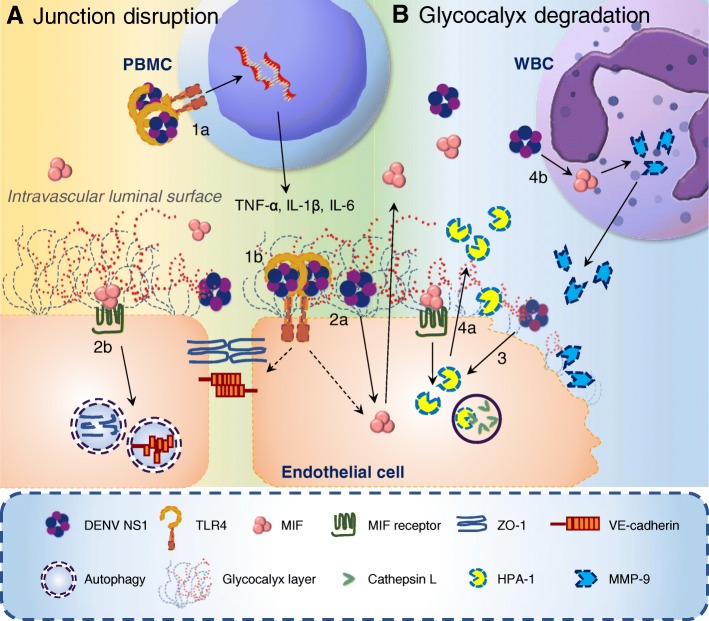
The possible mechanisms by which DENV NS1 causes vascular leakage. (1a) NS1 binding to TLR4 of PBMCs induces the expression and secretion of TNF-α, IL-1β and IL-6 cytokines, which may disrupt the tight junction, leading to vascular leakage [40]. (1b) NS1 binding to TLR4 or (2a) other molecules on endothelial cells induces the secretion of MIF [43]. (2b) MIF binding to its receptor on endothelial cells induces junction disruption through autophagic degradation of junction proteins such as ZO-1 and VE-cadherin [43]. (3) Binding of NS1 to endothelial cells also induces HPA-1 activation through cathepsin L, leading to endothelial glycocalyx degradation and vascular leakage [42, 49]. (4a) Additionally, NS1-induced MIF secretion is also involved in HPA-1 secretion of endothelial cells, and (4b) MMP-9 secretion of WBCs which can also contribute to endothelial glycocalyx degradation [65]

Shedding of the endothelial glycocalyx is related to activation of the heparan sulfate-specific glucuronidase HPA-1 [52, 56]. HPA-1 is synthesized as a 65-kDa non-active precursor that subsequently undergoes proteolytic cleavage to yield 8-kDa and 50-kDa subunits that heterodimerize to form an active enzyme. Activated HPA-1 enhances shedding of the transmembrane heparan sulfate proteoglycan syndecan-1 (CD138) and elevates the CD138 level in the bloodstream [57, 58]. In addition to HPA-1, metalloproteinase (MMP) family proteins are also important proteases that are capable of digesting the endothelial glycocalyx [59, 60] and increased MMP levels correlate with vascular leakage in DHF/DSS [61–64]. In 2017, Glasner suggested that DENV NS1-induced vascular leakage was independent of inflammatory cytokines, including TNF-α, IL-6 and IL-8, but was dependent on endothelial glycocalyx components, including cathepsin L and HPA-1 [42]. Recently, we further demonstrated that MIF is involved in DENV NS1-induced HPA-1 and MMP-9 secretion and degradation of the endothelial glycocalyx [65]. Taken together, the mechanisms of the vascular leakage that occurs during DENV infection may be very complex and may involve both the virus and the host immune response. The possible mechanisms by which DENV NS1 contributes to vascular leakage are shown in Fig. 1.

## The pathogenic roles of NS1 in coagulopathy and thrombocytopenia

In addition to vascular leakage, DENV NS1 may also contribute to severe dengue by disrupting coagulation. The NS1/thrombin complex was found in the sera of dengue patients, and binding of NS1 to prothrombin inhibited its activation, leading to a prolonged activated partial thromboplastin time [66]. However, whether NS1 is involved in thrombocytopenia is still unclear. It is known that LPS can induce platelet activation and potentiate platelet aggregation via TLR4/MyD88 signal transduction [67]. Since both NS1 and LPS can activate immune cells through TLR4, NS1 may induce platelet activation and enhance aggregation, possibly leading to over-destruction of platelets during dengue infection. Collectively, increasing evidence suggest that NS1 plays a crucial role in dengue pathogenesis by contributing to both vascular leakage and hemorrhage in dengue disease.

## DENV NS1 as a therapeutic target

### Current status of DENV treatment

Although many dengue patients only experience asymptomatic or mild signs of a flu-like illness followed by self-recovery within one week, some patients develop worse dengue symptoms that become life-threatening. To date, the treatment of dengue disease has been mostly supportive, and no licensed therapeutic drug is available. An effective drug against DENV infection is in great demand until a satisfactory vaccine becomes available. Because earlier observational studies stated that disease severity positively correlated with the viremia level and febrile phase during infections [68, 69], dengue researchers have put great effort into anti-viral approaches targeting different structural or nonstructural proteins. The inhibitory mechanisms of viral entry [70], pH-dependent viral fusion [71, 72], enzymes required for transcription/replication [73–77], and viral protein modifications [78] have been widely investigated. From this perspective, some off-patent drugs and antibiotics have also been tested for repurposing [79, 80], which is beneficial for shortening development time and costs. Although some drugs lead to significant viral reduction and provide effective anti-viral activity both in vitro and in animal models, in real world situations, these types of antiviral drugs face other limitations and challenges, probably due to untimely treatment in the clinic. People often enroll in clinical studies until their viremia declines during the later phase of illness. As a result, most cases, unfortunately, fail to meet the therapeutic endpoint measurements in clinical trials [71].

From another perspective, other studies have provided different insights into host modulation and immune regulation as therapeutic targets, which include targeting host dependency factors, host restriction factors, and host-mediated pathogenesis pathways [81]. In contrast to viral targets, host-targeting antiviral approaches are believed to avoid the rapid drug resistance or mutations that arise during viral evolution. Strategies targeting host factors have been widely reviewed using advanced mass screening approaches [82–84]. For instance, turn-on targets of antiviral responses are often reported to act as defensive therapies [85, 86]. Interferons exert diverse antiviral functions against viral replication, which can activate interferon-stimulated genes and related mechanisms. In addition, regulation of host metabolic pathways required for viral replication, such as glycolysis and autophagy, has been studied recently [87, 88]. However, these approaches can nonspecifically regulate the functions of different cells, and most of these approaches fail in clinical trials [89]. Recently, we found that minocycline, a semi-synthetic tetracycline-derivative antibiotic, attenuates DENV replication through inhibition of MIF secretion and autophagy formation both in vitro and in vivo [90]. In addition, minocycline treatment can prolong the survival of ICR suckling mice after DENV infection. Therefore, minocycline may modulate both virus replication and the host immune response. Further clinical trials of minocycline in dengue patients may help to verify its therapeutic protection against DHF/DSS.

### Antibody against NS1 as a therapeutic drug for dengue disease

In addition to small molecule drugs, antibody therapies are appreciated for their specificity against diseases. To date, many murine- or human-derived monoclonal antibodies have been developed to test their therapeutic effects against DENV infection in different studies. Antibodies targeting structural proteins, such as envelope and prM/M, have been characterized; these antibodies are termed “neutralizing antibodies” due to their blockage of viral entry and inhibition of viral attachment to host cells. However, safety issues and insufficient efficacy against all four DENV serotypes are often challenged due to the risk of ADE. The enhancement of viral infection by antibodies against DENV has been not only evaluated both in vitro and in vivo but also emphasized in a clinical cohort study [91]. Nevertheless, many researchers have identified mAbs that can neutralize DENV infection without ADE as a side effect. One study indicated that single-dose administration of human Ab513, which recognizes a linear epitope of envelope domain III, prevented DENV-induced thrombocytopenia in humanized mice with ADE [92]. Another study suggested that antibodies that recognized the envelope dimer epitope (EDE) were highly potent and broadly neutralizing antibodies [93, 94]. In addition, the human-derived mAb SIgN-3C with a LALA mutant abrogated the ADE effect and protected mice from lethal DENV-2 infection [95].

In contrast to Abs against structural proteins on the virion, such as E and PrM/M, which are effective only in the viremic phase, anti-NS1 Abs can provide different therapeutic mechanisms, by not only reducing viral propagation from infected cells in the early viremic phase but also attenuating NS1-induced disease development during the critical phase. In addition, because NS1 is not a viral structural protein, anti-NS1 Abs will not induce ADE. Indeed, anti-NS1 Abs can reduce viral replication by complement-dependent cytotoxicity (CDC) of infected cells and have been demonstrated in several flaviviruses, including DENV, in vitro and in vivo [96–99]. In addition, anti-NS1 Abs can block NS1-elicited pathogenic effects both in vitro and in vivo [38] and reduce DENV-induced mortality and morbidity in different mouse models (Table 1).

**Table 1:** Administration of Abs against DENV NS1 in different mouse models

Approach	Antibody administration	Challenge/routes	Mice	Outcomes	Reference
NS1 polyclonal antisera	500 l (i.p.) 24 h prior to challenge	100 LD50 of DENV2 (NGC) /i.c.	BALB/c	100% survival	[113]
Monoclonal ascitic fluid	1-10 mg/mouse (i.p.) 24 h prior to challenge	100 LD50 of DENV2 (NGC) /i.c.	BALB/c	50-93% survival	[113]
NS1 polyclonal antisera	300 μl (i.p.) cotreatment	NS1 (10 mg/kg) + 1×106 PFU of DENV2 (adapted strain D220)/ i.v.	Ifnar−/−C57BL/6	100% survival	[38]
Anti-NS1 mAb (1H7.4)	200 μg (i.p.) cotreatment	NS1 (10 mg/kg) +1×106 PFU of DENV2 (adapted strain D220)/ i.v.	Ifnar−/−C57BL/6	100% survival	[38]
Anti-DJ NS1 and anti-ΔC^a^ NS1 polyclonal Abs	50-150 μg/mouse (i.p.) 24 h after challenge	9×107 PFU of DENV2 (16681)/i.d.; 1×107 PFU of DENV2 (454009A)/i.v.	C3H/HeN	Reduce hemorrhage; rescue partial bleeding prolonged	[96, 114]
Anti-NS1 mAb (33D2)	100 μg/mouse (i.p.) 24 h after challenge	2×108 PFU of DENV1-4/i.d.; 4×107 PFU of DENV2 (454009A)/i.v.	C3H/HeN; STAT1-/-C57BL/6	80% survival; reduce viremia and NS1 antigenemia; reduce hemorrhage; rescue partial bleeding prolong	[97]
Anti-DJ^b^ NS1 polyclonal Abs	Two doses of 150 μg/mouse (i.p.) 24 and 48 h after challenge	1×107 PFU/mouse DENV2 (16681)/i.d.	STAT1-/- C57BL/6	Reduce mast cell degranulation, macrophage infiltration and chemokine production	[115]
Anti-NS1 mAb (2E8)	One dose of 50-150 μg/mouse (i.p.) either 1, 3, or 4 days after challenge	1×107 PFU/mouse	STAT1-/-C57BL/6	Reduce viremia and NS1 antigenemia; rescue partial bleeding prolong	[114]

^a^ΔC NS1: full-length DENV NS1 lacking the C-terminal amino acids (a.a.) 271-352

^b^Chimeric DJ NS1: consisting of N-terminal DENV NS1 (a.a. 1-270) and C-terminal Japanese encephalitis virus NS1 (a.a. 271-352)

**Fig. 2:**
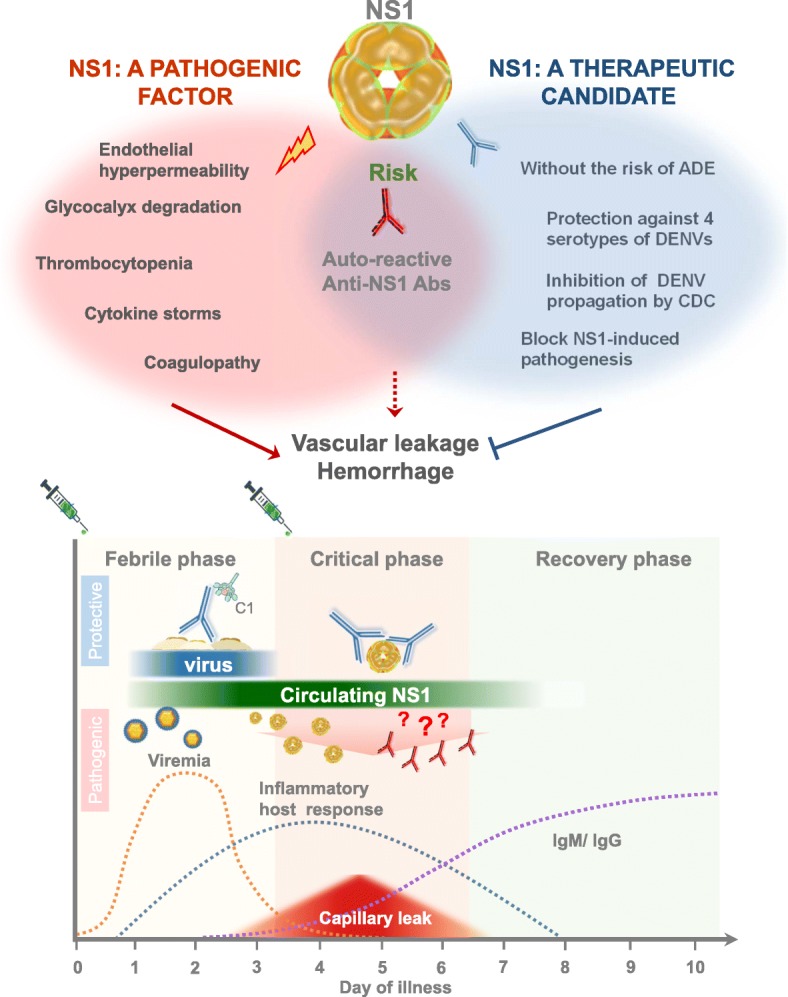
The possible pathogenic roles of DENV NS1 and its potential as a therapeutic target against DENV infection

However, as previously described, many anti-NS1 Abs can cross-react with host proteins; thus, antibodies generated from NS1 immune sera may contain cross-reactive Abs with undesired side effects [32, 100]. Therefore, we need to identify a subclass of protective anti-NS1 Abs that can block the activity of all four different dengue NS1 serotypes without cross-reactivity to host proteins. In our previous study, we analyzed a mAb against NS1 and identified Abs against a region of the NS1 wing domain that were protective against DENV infection in mice. Furthermore, the amount of these anti-NS1 Abs was inversely correlated with the severity of disease in dengue patients, indicating that the Abs were protective in patients [97]. Therefore, a mAb against this region of NS1 may represent an alternative choice for a therapeutic drug that is specific against DENV infection while avoiding the risk of ADE (Fig. 2).

## DENV NS1 as a vaccine candidate

### Current status of DENV vaccine development

The only licensed dengue vaccine currently available was developed by Sanofi Pasteur and has been approved in many countries. This live-attenuated tetravalent dengue vaccine (CYD-TDV; Dengvaxia) contains DENV E and prM proteins from the four serotypes in the yellow fever 17D backbone and has been used to induce preventive humoral and cell-mediated immune responses [101, 102]. Although CYD-TDV induced neutralizing Abs against all four DENV serotypes, this vaccine rendered only partial protection against serotype 2 DENV infection. In addition to the unequal efficacy of CYD-TDV against all four DENV serotypes, vaccination of this vaccine may induce non-protective Abs that enhance disease severity in persons who had not been exposed to dengue before. Indeed, it is reported that among children younger than nine years of age, the vaccine is associated with an increased incidence of hospitalization for severe dengue disease [103]. Therefore, the vaccination of seronegative individuals with Dengvaxia may enhance dengue disease severity but not protection due to ADE [104]. Conversely, some studies have suggested that the dengue vaccine fails to provide full protection, possibly due to the lack of T cell immunity elicited against nonstructural proteins or an NS1-induced protective immune response [105]. Collectively, these concerns make nonstructural proteins, including NS1, alternative options for dengue vaccine development.

### NS1 as a vaccine candidate against DENV infection

Both humoral and T cell-mediated cellular immune responses are critical for protection against DENV infection. Viral structural proteins have been regarded as potent targets to induce neutralizing Abs but are associated with the intractable issue of ADE. As a nonstructural protein, NS1 does not induce Abs against the virion. NS1 is the only nonstructural protein of DENV that can be both anchored on the surface of infected cells as a membrane-associated homodimer and released from infected cells into circulation as a hexamer. These properties of NS1 make it able to trigger both cellular and humoral immunity. Because NS1 is exposed on infected cells, the complement cascade can be triggered by NS1-bound anti-NS1 Abs [106]. Complement activation can lyse infected cells via CDC and eventually reduce viral titers through suppression of viral propagation. Studies have shown that immunization with DENV NS1 via different approaches can induce protective immune responses against DENV infection in mice, as shown in Table 2.

**Table 2:** Different NS1-based vaccine strategies in mouse models

Vaccine type	Approaches	Challenge/routes	Mouse	Outcome	Reference
Protein	Full length NS1 + CFA adjuvant	Lethal amount of DENV2 (NGC) from suckling mouse brain/i.c.	CD1	88% survival; 35% reduction in morbidity	[98]
	Recombinant vaccinia virusexpressed NS1	100 IC50 of DENV4 (H241) or DENV2 (NGC)/i.c.	BALB/c	63-100% survival	[116]
	rEC204-NS1N65 –protein A	100 LD50 of DENV2 (NGC)/i.c.	BALB/c	100% survival	[117]
	rNS1+ LTG33D adjuvant	4.32 log10 PFU of DENV2 (NGC)/i.c.	BALB/c	50% survival; 10% reduction in morbidity	[118]
	ΔC NS1# + CFA adjuvant	9×107 PFU of DENV2 (16681)/i.d.	C3H/HeN	66% reduction in hemorrhage; rescue partial bleeding prolong	[96]
	Chimeric DJ NS1## + CFA adjuvant	9×107 PFU of DENV2 (16681)/i.d.	C3H/HeN	66% reduction in hemorrhage; rescue partial bleeding prolong	[96]
	Full DENV 1-4 NS1 + MPLA/AddaVax adjuvant	1×107 of DENV2 (adapted strain D220)/i.v.	Ifnar−/−C57BL/6	60-100% survival; reduce viremia and NS1 antigenemia	[38]
Subunit peptide	Modified NS1-WD^a^+ CFA adjuvant	2×108 PFU of DENV1-4/i.d.; 4×107 PFU of DENV2 (454009A)/i.v.	C3H/HeN; STAT1-/- C57BL/6	100% survival; reduce viremia and NS1 antigenemia; 70-90% reduction in hemorrhage; rescue partial bleeding prolong	[97]
	pD2NS1/pD2NS1+ pIL-2	5×106 -107 PFU of DENV2 (PL046)/i.v.	C3H	50-80% survival; 70-80% reduction in morbidity	[119]
DNA vaccine	pcTPANS1^b^	4.32 log10 PFU of DENV2 (NGC)/i.c.	BALB/C	100% survival	[120, 121]
	pcENS1^c^	4.32 log10 PFU of DENV2 (NGC)/i.c.	BALB/C	86.7% survival; 60% reduction in morbidity	[122]

^a^NS1-WD: wing domain region of NS1

^b^TPA: human tissue plasminogen activator; a secretory signal sequence.

^c^pcENS1: encoding the C-terminal E protein plus the full NS1 region

However, immunization of mice with full length of DENV NS1 can induce Abs cross-react with host proteins have also been demonstrated by different groups and these cross-reactive Abs can cause pathological effects both in vitro and in mice [107–109]. Although the contributions of these cross-reactive Abs in dengue pathogenesis are still under debate, the potential side effects induced by vaccination with full-length NS1 should be avoided in dengue vaccine design. Since most of these cross-reactive Abs recognize the C-terminal region of NS1. [110], different approaches have been applied to prevent the induction of cross-reactive Abs by NS1 immunization. For instance, NS1 lacking its C-terminus (ΔC NS1) was used to immunize mice; the results showed that ΔC NS1-elicited Abs provided better protection against DENV infection than immunization with full-length NS1 [96]. However, in addition to the C-terminus, other regions of NS1 also show molecular mimicry to host proteins and can elicit auto-reactive Abs against endothelial cells and coagulation factors [111, 112]. To avoid the risk of the induction of cross-reactive antibodies by NS1 immunization, a short modified NS1 peptide containing an 11-a.a. conserved wing domain region of NS1 was designed; this peptide modified the critical pathogenic amino acids to reduce cross-reactivity but maintain immunogenicity. Importantly, both active immunization with the modified NS1 peptide and passive transfer of polyclonal Abs against the modified NS1 peptide provided protection against DENV in a hemorrhagic mouse model and a lethal infection mouse model [97].

## Conclusions

Collectively, this review discusses the critical pathogenic roles of NS1 in dengue pathogenesis. NS1 is considered a unique “viral toxin” in dengue disease. Therapeutic approaches and vaccine development targeting NS1 may provide different opportunities to combat dengue disease.

## Abbreviations

CFR: Case-fatality rate
CXCR: CXC chemokine receptors
DENV: Dengue virus
DF: Dengue fever
DHF: Dengue hemorrhagic fever
DSS: Dengue shock syndrome
E: Envelope protein
ER: Endoplasmic reticulum
GAG: Glycosaminoglycan
IL: Interleukin
iNOS: Inducible NO synthase
MIF: Macrophage migration inhibitory factor
MMP: Matrix metalloproteinase
NO: nitric oxide
NS1: Nonstructural protein 1
PBMCs: Peripheral blood mononuclear cells
prM: Pre-membrane protein
TLR: Toll-like receptor
TNF: Tumor necrosis factor
